# Bacterial community diversity on in-shell walnut surfaces from six representative provinces in China

**DOI:** 10.1038/s41598-017-10138-y

**Published:** 2017-08-30

**Authors:** Lihui Zhang, Shaojin Wang

**Affiliations:** 1 College of Mechanical and Electronic Engineering, Northwest A&F University, Yangling, Shaanxi 712100 China; 2 Department of Biological Systems Engineering, Washington State University, Pullman, WA 99164-6120 USA

## Abstract

Walnuts (*Juglans regia*) have been associated with foodborne illness outbreaks in recent years. Thus, the purpose of this study was to investigate the distribution of bacteria on in-shell walnut surfaces from six representative provinces in China. The bacterial populations on walnut surfaces were investigated by high-throughput sequencing based on the bacterial 16 S rRNA hypervariable region V4. Twenty-eight samples were collected from fourteen regions in six provinces and harvested in different periods (the fresh in 2016 and the old in 2015). *Proteobacteria* was the most dominant phylum in all samples except for XJ1. In XJ1, and the most abundant phylum was *Cyanobacteria*, which also accounted for a large proportion of the abundance in YN1, YN11, XJ2 and SC11. In addition, *Firmicutes* and *Actinobacteria* were also the abundant phyla in the given samples. Some genera belonging to the opportunistic pathogens were detected, such as *Pseudomonas*, *Acinetobacter*, *Burkholderia* and *Bacillus*. The results revealed that the composition and abundance of bacterial consortiums on walnut surfaces varied among the geographical sites where they were harvested. Moreover, the storage time of samples also had impact on the abundance of bacteria. This study may provide a better understanding of the bacterial communities’ diversity on in-shell walnut surfaces.

## Introduction

Walnuts (*Juglans regia*) contain many nutrients, including proteins, fatty acid, vitamin E, mineral, folate, melatonin, numerous polyphenols, etc.^1, 2^. The walnut is rising in popularity for its beneficial effects to the brain, the blood lipid profile, cardiovascular disease, diabetes and so on^3, 4^. For instance, walnuts can increase in human inferential verbal reasoning^5^ and have been associated with a reduced risk for cancer^6, 7^. In China, there are a large number of walnuts to export every year^8^. The production of in-shell walnuts reached 1.06 million metric tons in 2016^9^.

The fleshy husks of walnuts are removed shortly after harvest, and then the in-shell walnuts are dried naturally or with forced air. Finally the walnuts may be stored before shelling or other processing. Due to the unique harvesting methods, the walnut surface is unavoidably infected by a number of microorganisms in the environment, and most likely to be re-infected during the process of storage, transportation and sales. These microorganisms may include some foodborne pathogens, such as *Aspergillus*, *Salmonella*, *Shigella*, *Listeria monocytogenes*, and *Escherichia coli O157:H7* ^10^, which can cause foodborne illness and even death in humans. Additionally, some bacteria and fungi can produce toxins, which are hardly to control during the processing. The fruits and nuts account for 7% of the total foodborne outbreaks^11–13^, resulting in serious concerns from food industry and local commniuties. Especially for tree nuts, such as walnuts, almonds, pistachios, and peanuts, there have been reported some outbreaks and recalls caused by bacterial pathogens^14–16^. Some studies have reported that these bacterial pathogens are able to survive in low-water activity foods for long-term storage^17, 18^. Blessington *et al*. also found that foodborne pathogens could survive not only in walnut kernels but also on in-shell walnut surfaces for a long time during storage^19, 20^. In China, since there are a few reports about nut-induced disease outbreaks, it is necessary to investigate the existence and distribution of bacterial pathogens on nut shells.

Currently, conventional culture-based assay and molecule-based methods are mainly used to detect these foodborne pathogens. The culture-based detection methods are time consuming and complex, and some non-culturable pathogens may be ignored. The molecule-based methods, such as PCR, qPCR, and mPCR, are frequently used because of time-efficient and labor-saving^21, 22^. While these methods also have limitations, they cannot reveal the diversity of overall pathogens in the samples. Nowadays, high-throughput sequencing (HTS) technology, also known as “next-generation” sequencing technology, can be used to characterize microbial community composition of complex environmental ecosystems. HTS technology has been applied in many fields to investigate the microbial diversity, including food samples^23, 24^, plants^25^, soil and water samples^26–30^.

The shells of nuts may prevent some direct contamination from the surface to the inner kernel^31^. Walnut kernels are completely wrapped by shells, thus most of the microorganisms are attached to the shells. Thin-skinned walnuts are easily broken, which may make the kernels being infected. In China, most of raw walnuts are directly consumed and usually added to foods as main gradients, resulting in great risks for consumers to expose to the potential pathogens. Thus, it is required to investigate the microbial diversity on walnut shells and provide basic information on target pathogens for designing effective pasteurization processes.

The objectives of this study were (1) to analyze the bacterial diversity on the surface of in-shell walnuts located in major producing regions in China, (2) to investigate the possible bacterial pathogens on the in-shell walnuts surface, and (3) to compare the changes of bacterial community among these samples in different regions and storage times.

## Results

### OTU-based analyses: richness and diversity indexes

**Figure 1:**
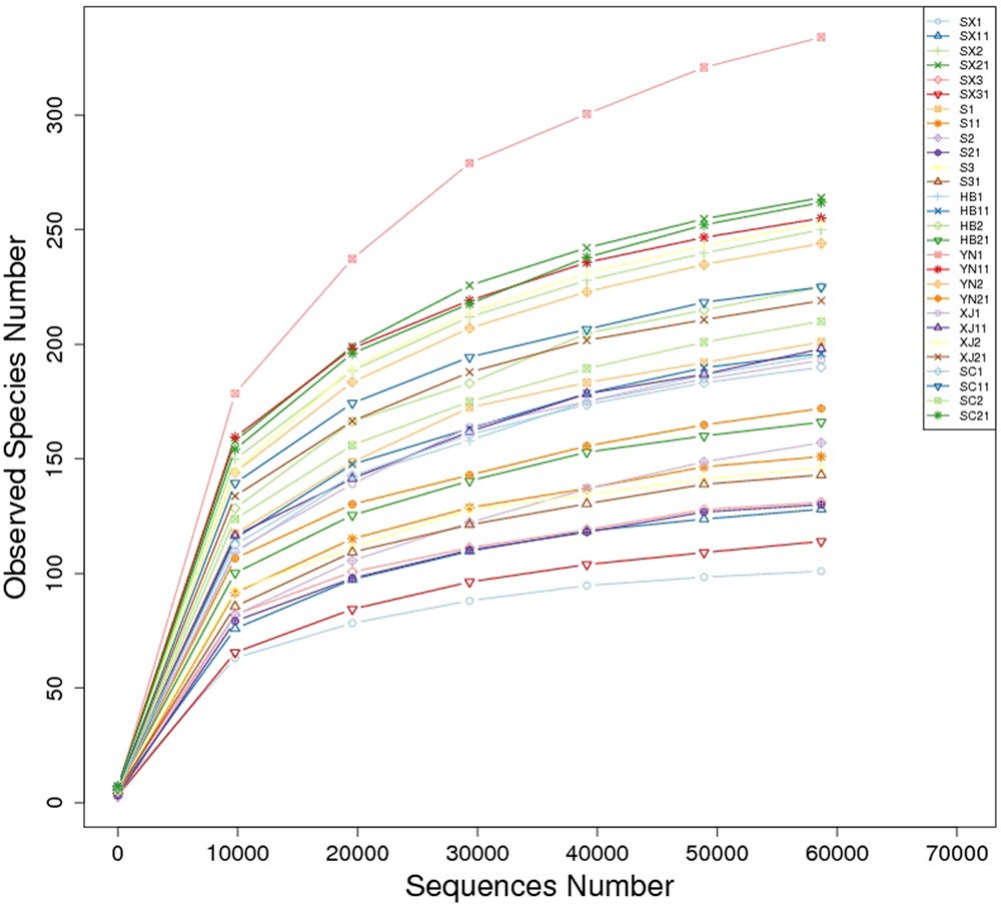
Rarefaction curves of the twenty-eight samples.

A total of 1,867,565 effective tags obtained by HTS were clustered into operational taxonomic units (OTUs) based on 97% identity. An average of 224 OTUs were obtained, which varied from a minimum of 111 to a maximum of 369 OTUs per sample. In alpha diversity analysis, rarefaction curves, Chao1, and Shannon’s index were also generated to reflect community richness and diversity. All the indexes showed that the differences among samples did not follow a specific trend according to geography and times. The highest species richness occurred in YN1 according to rarefaction curves analysis of OTUs in the samples, which were confirmed by Chao1 index (Table 1). The trend of rarefaction curves suggested a sufficient sampling of the microbial communities (Fig. 1). Good’s coverage estimate showed high values, all above 99.9% (Table 1), indicating that the sequence numbers per sample were high enough to obtain complete coverage of the bacterial diversity.

**Table 1:** Summary of diversity indices for all studied samples.

Group	Sampling regions	Samples	Effective Tags	No. of OTUs	Shannon	Chao1	Simpson	Good’s coverage	ACE	Numbers of initial colonies (log CFU/mL)
Z1	Yangling	SX1	64,648	111	3.25	103.87	0.84	100.00%	108.65	2.47 ± 0.02
		SX11	71,135	162	2.90	135.78	0.80	100.00%	142.84	2.46 ± 0.01
	Shangluo	SX2	63,674	274	4.07	285.42	0.89	99.90%	293.21	3.40 ± 0.01
		SX21	70,765	316	4.02	287.5	0.85	99.90%	294.04	3.41 ± 0.02
	Yanan	SX3	67,696	152	3.11	150.12	0.81	100.00%	153.64	3.10 ± 0.02
		SX31	74,798	135	2.12	129.79	0.61	100.00%	134.66	2.87 ± 0.04
Z2	Fenyang	S1	69,111	235	4.18	230.12	0.91	99.90%	243.33	2.47 ± 0.01
		S11	66,884	177	2.80	174.62	0.71	99.90%	180.70	3.45 ± 0.02
	Zuoquan	S2	65,369	172	1.50	208.00	0.36	99.90%	217.57	2.91 ± 0.02
		S21	58,762	149	2.14	147.10	0.64	100.00%	150.33	2.98 ± 0.02
	Licheng	S3	60,511	167	2.76	159.14	0.73	100.00%	161.47	2.93 ± 0.01
		S31	58,779	167	3.03	154.50	0.79	100.00%	157.88	3.12 ± 0.02
Z3	Handan	HB1	66,109	226	3.07	230.69	0.78	99.90%	233.43	3.36 ± 0.03
		HB11	59,317	224	2.81	212.58	0.70	99.90%	224.48	2.51 ± 0.02
	Yanshan	HB2	68,866	268	3.67	257.24	0.87	99.90%	266.80	3.26 ± 0.01
		HB21	68,471	196	3.26	182.73	0.83	99.90%	185.33	2.45 ± 0.02
Z4	Dali	YN1	66,955	217	3.95	372.73	0.89	99.90%	390.80	3.48 ± 0.01
		YN11	62,216	268	4.33	275.00	0.89	99.90%	280.90	3.44 ± 0.01
	Dayao	YN2	63,206	240	3.99	273.68	0.89	99.90%	279.32	3.49 ± 0.01
		YN21	67,420	308	3.26	195.78	0.81	99.90%	210.25	3.43 ± 0.01
Z5	Aksu	XJ1	69,596	207	2.71	210.03	0.67	99.90%	215.49	3.45 ± 0.01
		XJ11	72,364	230	3.94	237.00	0.90	99.90%	260.28	3.09 ± 0.01
	Hetian	XJ2	66,670	286	3.87	286.60	0.89	99.90%	289.19	3.31 ± 0.02
		XJ21	69,273	255	3.50	248.69	0.82	99.90%	253.76	3.26 ± 0.01
Z6	Guangyuan	SC1	68,211	369	3.95	211.17	0.90	99.90%	223.75	2.45 ± 0.01
		SC11	65,657	282	3.78	247.45	0.85	99.90%	252.48	2.46 ± 0.01
	Ganzi	SC2	69,974	274	3.53	254.20	0.82	99.90%	264.86	2.44 ± 0.01
		SC21	71,128	197	4.21	284.61	0.90	99.90%	300.92	3.13 ± 0.01

Microbial counts of walnuts samples were the means of two replicates, expressed as log CFU/mL. All the samples with “1, 2, and 3” represent the walnuts harvested in 2015; all the samples with “11, 21, and 31” represent the fresh walnuts in 2016.

### OTU distribution from Venn plots and flower plots

**Figure 2:**
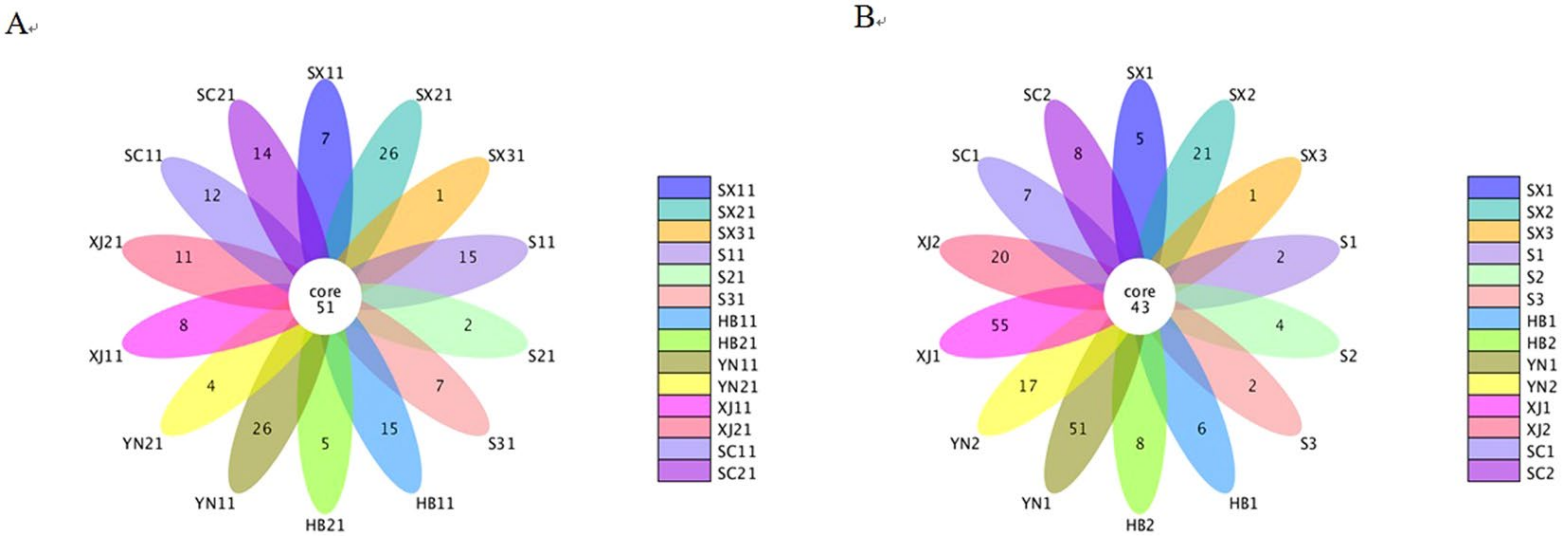
Flower diagram of the OTUs in the twenty-eight samples (**A**) the fresh samples in 2016 and (**B**) the samples in 2015 in different regions. The numbers inside the diagram indicate the numbers of OTUs.

Venn plots (samples <5) and flower plots (samples >5) were used to show the shared and unique OTUs in each plotted group. These plots more intuitively showed the unique and overlap of the OTU composition of the samples. The fresh samples (2016) shared 51 OTUs, which were slightly higher than those (43 OTUs) in 2015. The numbers of unique OTUs changed obviously among samples of Yunnan and Xinjiang provinces in 2015 and 2016 (Fig. 2). The XJ1 sample had the highest unique OTU numbers. According to the Venn plots or flower plots of the samples within the same province, the samples HB11, SC11 and SC21 had higher OUT numbers than those of HB1, SC1 and SC2 samples (see Supplementary Fig. S1).

### Bacterial community analysis

The initial numbers of colonies from walnut surfaces were detected simultaneously. The initial colonies in all samples were < 3.5 log (Table 1). The morphology of the colonies was diverse.

**Figure 3:**
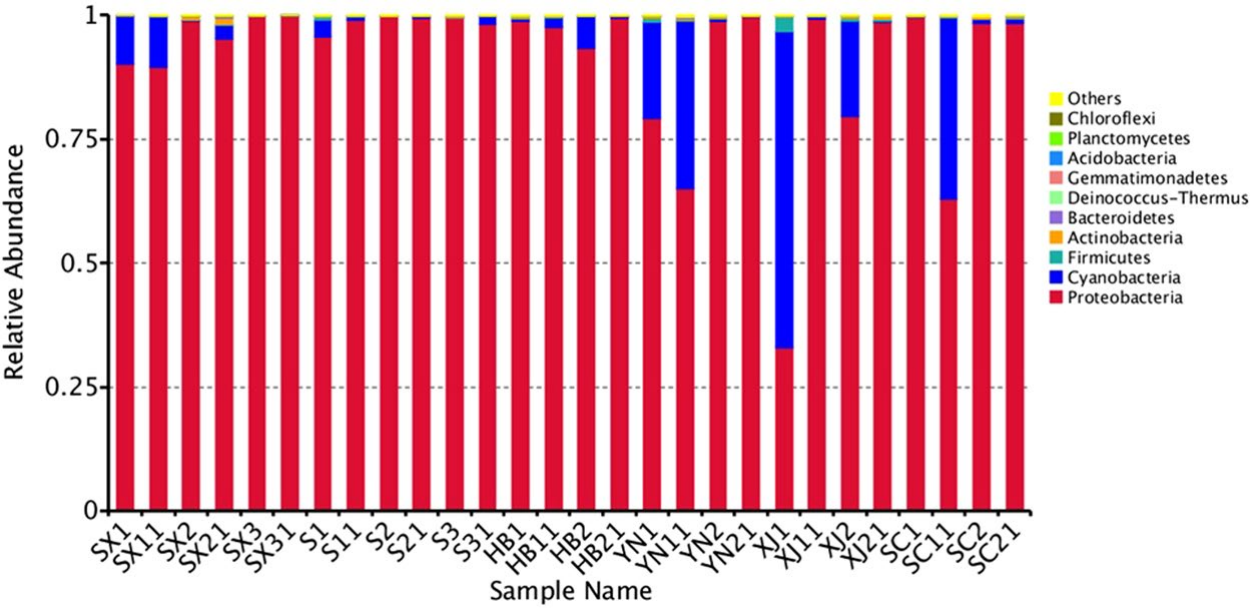
Composition of bacterial community at phylum level. The top ten phyla are shown, and the other phyla are included as “Others”.

For high-throughput sequencing analysis, all the obtained sequences were taxonomically classified from phylum to genus. The twenty-eight samples appeared to have very similar 16 S rRNA profile in the phylum level. The ten largest phylum are shown in Fig. 3. *Proteobacteria* dominated the observed sequences at phylum level. *Cyanobacteria* phylum accounted for a large proportion of the sequences in XJ1 (63.59%), XJ2 (19.13%), SX1 (9.78%), SX11 (10.17%), HB2 (6.55%), YN1 (19.44%), YN11 (33.80%) and SC11 (36.38%). In addition, *Firmicutes* were high in XJ1 sample, accounting for 3.02%. *Actinobacteria* was the third largest phylum (1.32%) in the SX21 sample. Within *Proteobacteria*, *Gammaproteobacteria* was the most dominant group (54.9–99.4%) in these samples except for XJ1. *Alphaproteobacteria* and *betaproteobacteria* occupied a large part of the relative abundance in all samples. *Enterobacteriales* were the most abundant order within the *Gammaproteobacteria*. However, a much higher proportion of the unidentified sequences existed in all the samples. The proportion of *Pseudomonades* in all the samples was from 3.69% to 28.5%. The other seven dominant orders included *Rickettsiales*, *Xanthomonadales*, *Sphingomonadales*, *Burkholderiales*, *Rhizobiales*, *Lactobacillales*, and *Rhodobacterales*.

**Figure 4:**
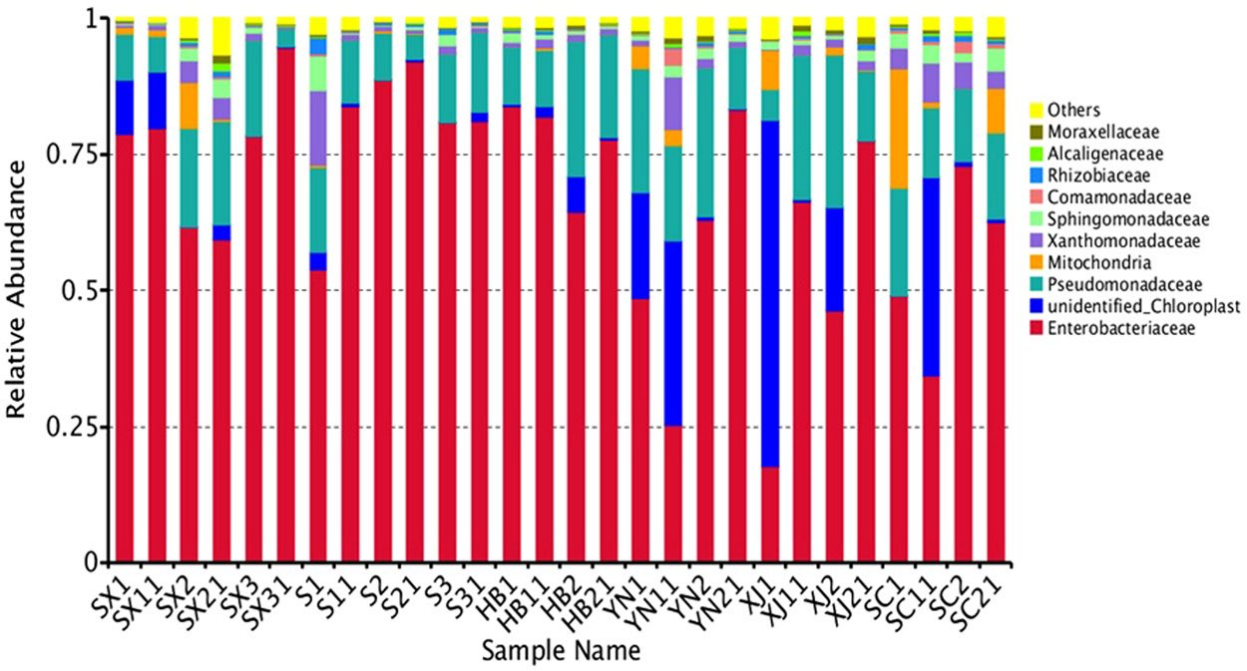
Composition of bacterial community at family level. The top ten families are shown, and the other phyla are included as “Others”.

Diversity of the samples became more evident at family level classification. *Enterobacteriaceae* were found in all samples with the relative abundance from 17.7% to 94.6% at family level. Microorganisms assigned to unidentified bacteria existed with a much higher proportion in YN1, YN11, XJ1, XJ2, and SC11. *Pseudomonadaceae*, *Mitochondria* and *Xanthomonadaceae* were comprised of the main remaining diversity for the samples at family level (Fig. 4).

**Figure 5:**
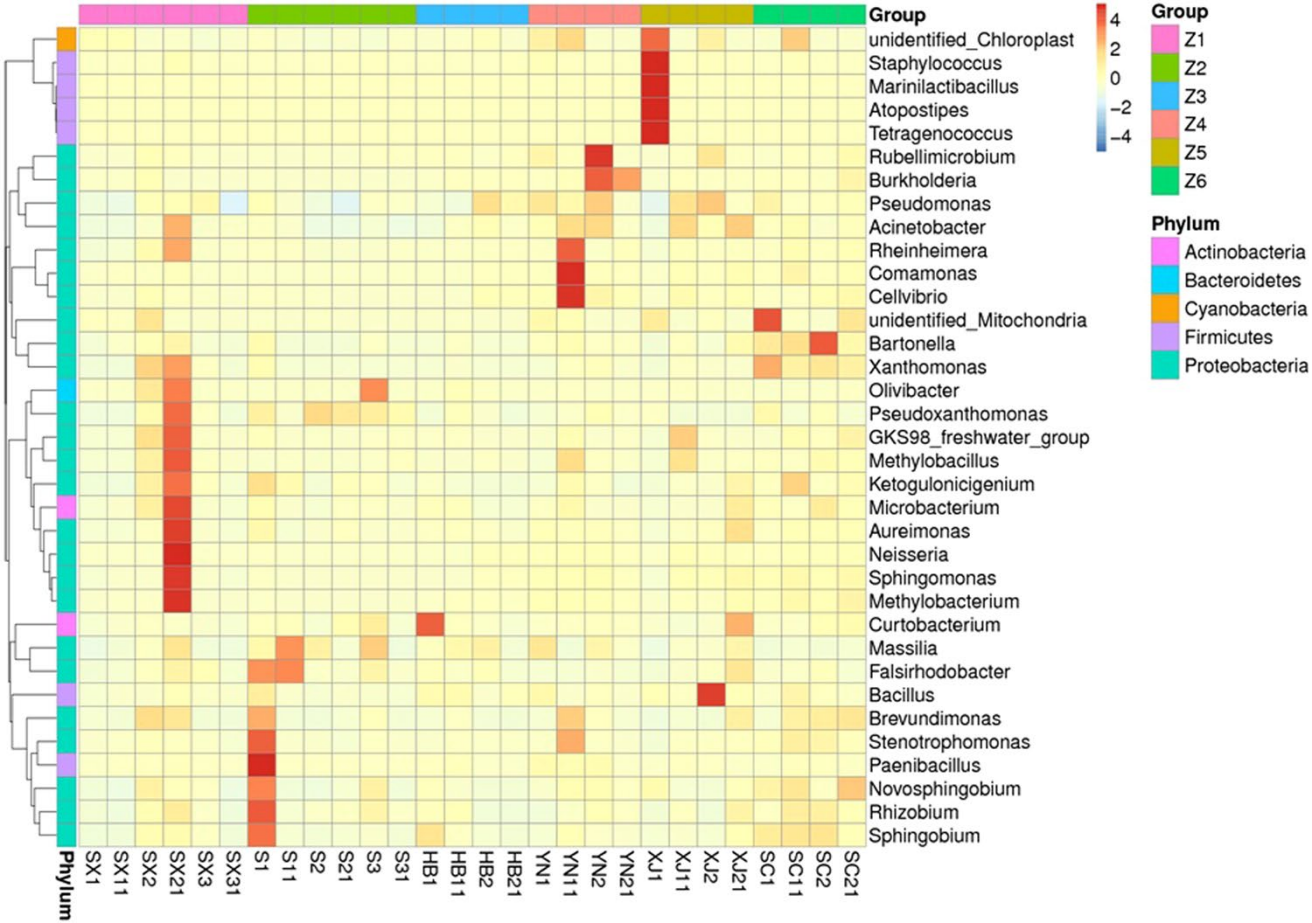
Heat map of the top 35 genera for all the samples. The color intensity with the legend at the right of the figure indicates the relative values for each genus.

At the genus level, “Others” (the sum of the relative abundance of the genera except for the top ten) occupied a large percentage. The most abundant genus was *Pseudomonas* except for unidentified sequences. *Stenotrophomonas*, *Novosphingobium*, *Rhizobium*, *Xanthomonas*, *Sphingomonas*, and *Acinetobacter* were also abundant relative to other genera. The heat map was used to analyze the variety of the abundance of most genera in the twenty-eight samples (Fig. 5). *Staphylococcus* existed in many samples. Some possible pathogens were also found, such as *Pseudomonas*, *Acinetobacter*, *Burkholderia* and *Bacillus*.

### Beta diversity analysis

**Figure 6:**
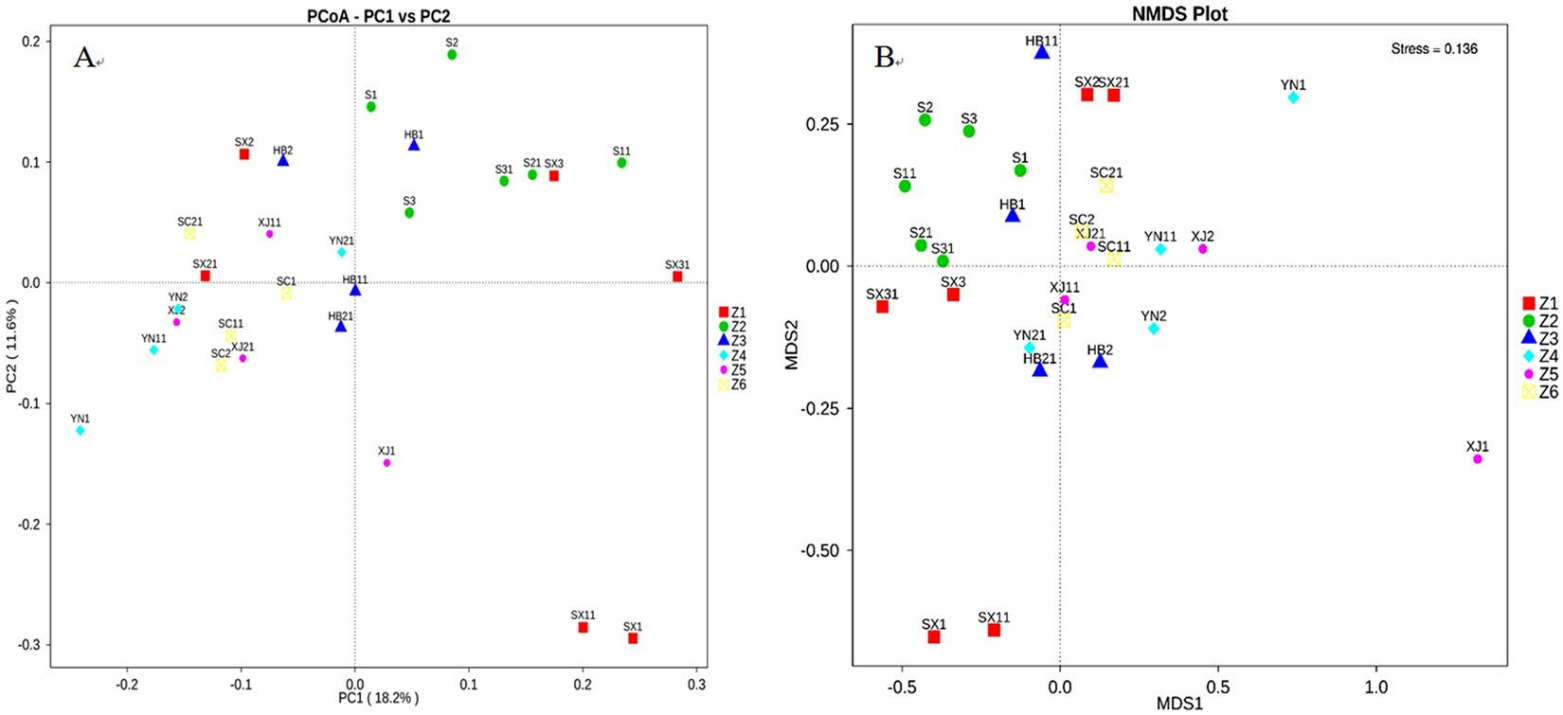
PCoA (**A**) and NMDS plot analysis (**B**) based on un-weighted Unifrac metrics for all samples.

**Figure 7:**
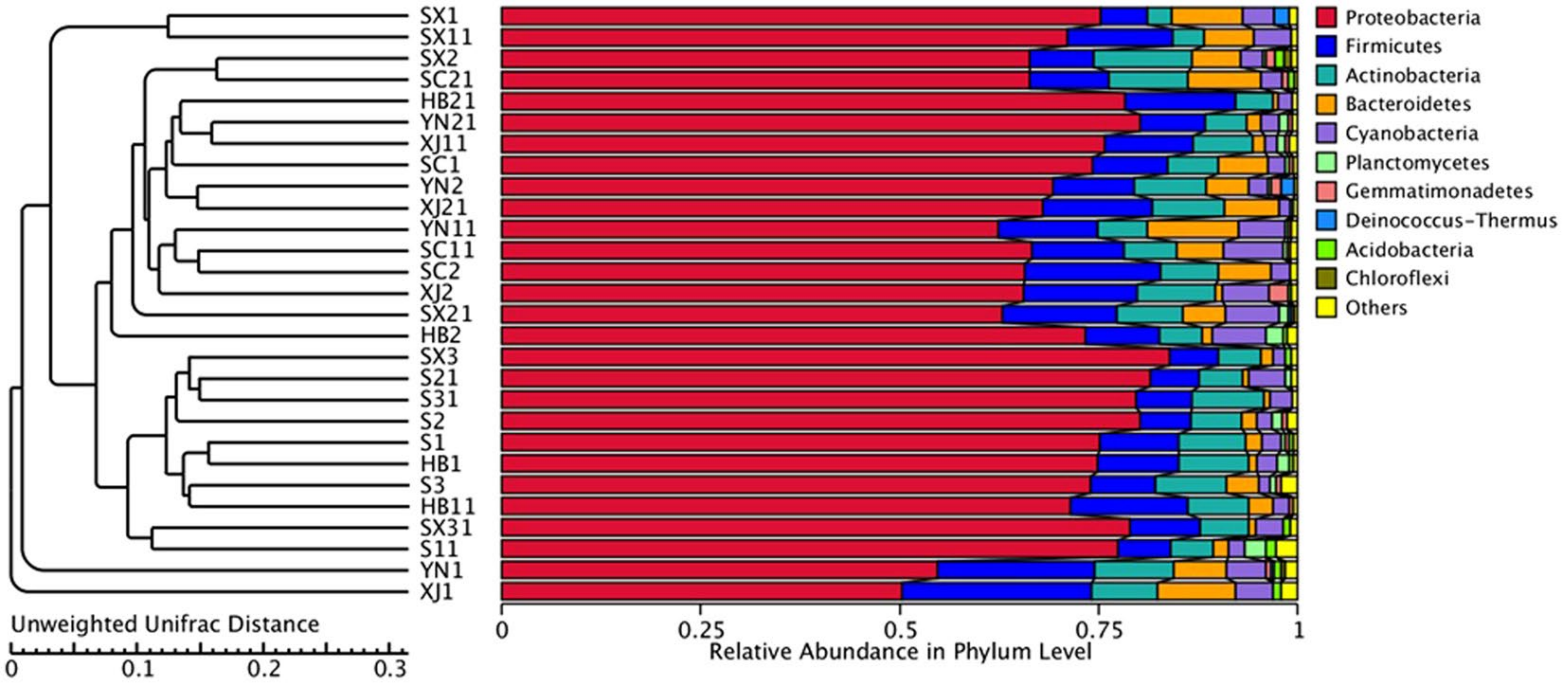
UPGMA clustering tree based on un-weighted Unifrac distance. The relative abundances of the top ten phyla in all samples are indicated, and the rest phyla are indicated as ‘Others’.

Principal Coordinates Analysis (PCoA) was performed to visualize and compare the relationship of the microbial communities among the twenty-eight samples. Based on PCoA of un-weighted UniFrac distance (Fig. 6A), the bacterial communities of the samples in Z2, Z3 and Z6 were more closely grouped according to PC1 (53.09%) and PC2 (28.37%). While the samples with different storage times from the same area were not grouped together. For example, S11 was closer to SX31 than S1. In Z1, the samples of the three regions were separated from each other, whereas samples in the same area at different times were closer with each other. XJ1 in Z5 was separated from all the other samples. The samples in Z6 were also separated from each other especially for the sample YN1. This result was consistent with Non-Metric Multi-Dimensional Scaling (NMDS) (Fig. 6B), which could better reflect the nonlinear structure of ecological data. The un-weighted UniFrac distance cluster analysis was used to show the similarity between different samples. The results of UPGMA clustering tree confirmed those of PCoA and NMDS (Fig. 7).

## Discussion

This study provides the diversity of bacterial community on the in-shell walnut surface based on the high-throughput sequencing method. *Proteobacteria*, *Firmicutes*, *Bacteroidetes*, and *Acinetobacteria* were the dominant phyla in the taxonomic groups of all the samples. These phyla present in many environments and food samples, although the relative abundances vary in these samples^32–35^. *Gammaproteobacteria*, *Alphaproteobacteria* and *Betaproteobacteria* were the three main classes within *Proteobacteria*. These classes were also found in the tomato fruit surface and some plant species^36, 37^. *Cyanobacteria* are ubiquitous microorganisms, and may synthesize algal toxins^38, 39^. *Cyanobacteria* existed in all the samples with an obvious change in abundance in this study. *Cyanobacteria* also constituted significant portion in soil microbes^40^. Thus the existence of *Cyanobacteria* might be due to soil or water sources in different areas. *Actinobacteria* are minor microbes in soils and play an important role in natural ecosystems^26^. *Actinobacteria* were also found in some food materials like cocoa beans^41^. *Acidobacteria*, *Bacteroidetes* and *Chloroflexi* were ubiquitous in natural environments^26, 42^ and were in the top ten phyla in this study. The walnut surfaces might be infected by these bacteria through the soil and water in the environment during the walnut harvesting and processing.

There was a high proportion of bacteria identified as the family *Enterobacteriaceae* in this study. Some important human pathogenic bacteria are included in this family and they can spread easily among humans. This family had high adaptability on the walnut surface according to the results. Thus it might be associated to the higher risk of disease outbreaks associated with walnuts. However, sequences for major foodborne pathogens genera were not found.

The identification at genus level based on the V4 region of the 16 S rDNA presented a more diverse bacterial community on the in-shell walnut surface. Genera here found were unlike those in some fermented foods and seafood^23, 34, 43, 44^. The distribution of the bacteria was more similar to that in soil and water samples at genus level. This might be related to the environment that walnuts are exposed. *Pseudomonas* and *Stenotrophomonas* are both widely distributed in soil and water. Walnuts are the field-grown crop and may be completely exposed to the external environment during processing, thus they are easy to be infected by these two genera. *Stenotrophomonas* is an opportunistic pathogen genus although it may have beneficial effects on plant growth^45^. *Sphingomonas* were found to be more abundant in SX21 than other samples and also observed in the abalone samples^44^. *Acinetobacter* can cause disease under certain conditions and mainly exist in water and soil. This genus appeared in all the samples in this study and had also been found in raw milk^33^. The *Burkholderia* genus has a great threat to human health, even though it may be beneficial to plants^46^. In our study, *Burkholderia* was the relatively lower abundant genus found in twenty-four samples. The genus *Staphylococcus* was also identified with low abundance in the samples, since it is a potential pathogen genus and may sometimes lead to safety hazards. The presence of these potential pathogens genera suggests that the walnuts industry should have a standardized management to meet food safety requirements. The presence of the unidentified-chloroplast in the samples was consistent with *Cyanobacteria*, which may have impacted on the taxa of bacteria^47^.

From the alpha diversity analysis and Beta diversity analysis, we can know that the microbial richness differs in different samples. The cluster analysis suggests that the geographic conditions and storage time have a certain impact on the abundance of bacteria on these samples. The differences among walnut surface environments appear to be linked to the plant conditions in different locations. These results in this study were similar to those of the tomato surface^37^. Zhou *et al*. also pointed that the *cecal microbiota* of Tibetan chickens differed due to geographic location^35^. The abundance of bacterial on in-shell walnut surface with different storage times varied even though they were harvested in the same region. The effect of storage time on the abundance of bacteria was more obvious in XJ1 and XJ11, YN1 and YN11. This might be caused by storage conditions like temperature and humidity^18, 44^.

In addition, the walnut varieties may be linked to the diversity of bacteria because of their shells. Some samples may be more easily attached and result in the bacteria being hardly washed away by water according to their uneven shells. The study of the spinach confirmed that the leaf surface topography had impact on the spinach epiphytic bacterial community^48^. Some pretreatment processes for the walnuts like drying, sterilization and bleach may also impact the bacteria abundance. These treatments could affect the microbial growth and survival especially for some major pathogens^49–51^.

## Conclusion

It is essential to investigate the bacterial diversity on in-shell walnut surface for food safety. This study showed the bacterial diversity on in-shell walnut surface from different locations and storage times based on high throughput sequencing technology. Similar to many materials, *Proteobacteria* accounted for a large proportion of abundance in the phylum in all the samples. While different from some food materials at genus level, the distributions of bacterial communities on in-shell walnut surface were similar to those in some materials like soils, waters or some field-grown crops. The taxonomies of the bacterial communities of most samples were similar, while the abundances of the taxa were different among these samples. The composition of the bacterial community on in-shell walnut surfaces varied slightly. However, the abundance of these bacteria varied with locations and storage times. The results of the representative samples can provide an important contribution to the understanding of the bacterial communities on in-shell walnut surface. The distribution of bacteria on in-shell walnut surfaces also provided basic information on target pathogens for pasteurization. In this study, we just collected samples in six main producing provinces without considering the varieties, storage conditions and other factors. To better study the factors affecting microbial community on in-shell walnut surfaces, further studies need to be conducted to cover more walnut varieties, the storage conditions, and pre-treatment methods.

## Materials and Methods

### Sample collection

**Figure 8:**
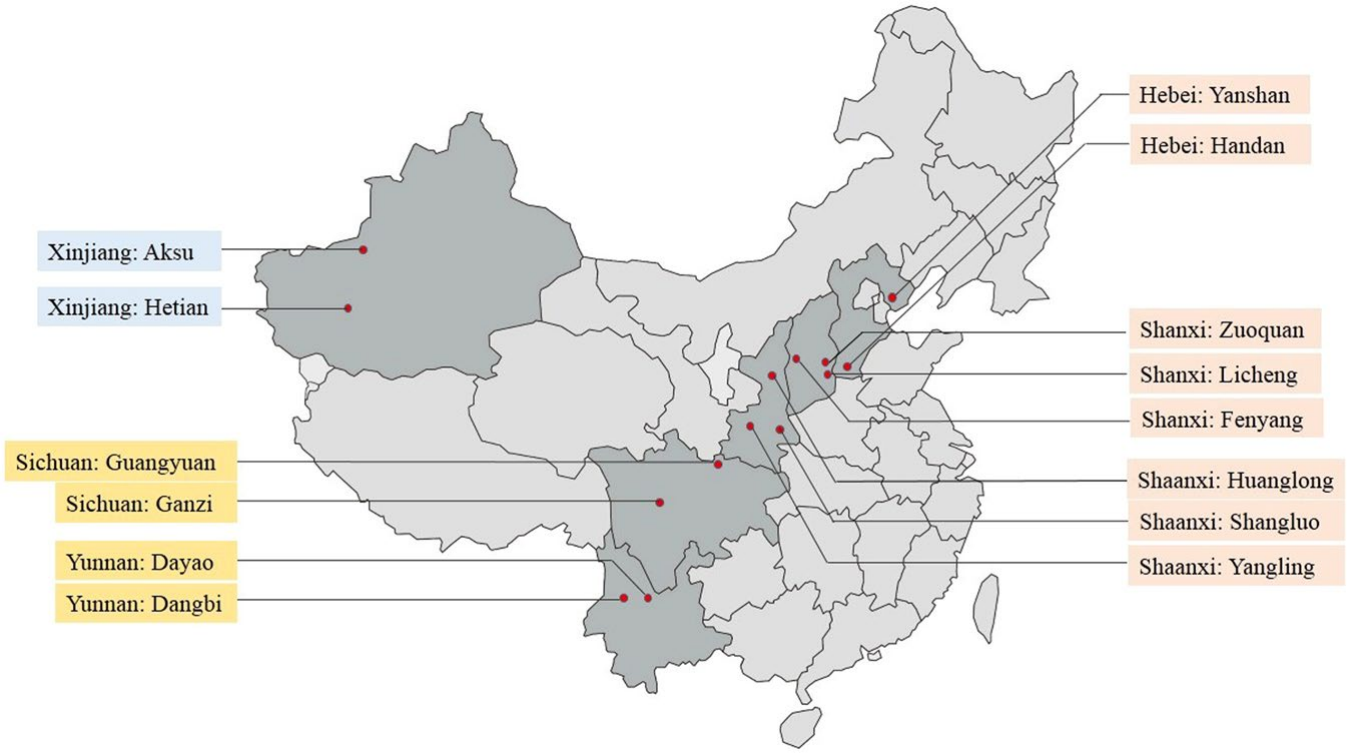
Sampling sites of walnuts (the original figure was available at http://www.map1000.com/, and then updated with mapinfo8.5). Samples were obtained from fourteen regions including the fresh walnuts in 2016 and the stored walnuts harvested in 2015.

In-shell walnuts were collected from fourteen areas, which were major walnut-producing areas in China (Fig. 8). The samples were collected from May to October in 2016, including the fresh walnuts harvested in 2016 and the stored samples harvested in 2015 (Table 1). The storage times of the samples harvested in 2015 were not more than one year. These samples were collected from local suppliers or markets and were all stored in the warehouse before the sale. The walnut samples from the same province were put into the same group (Table 1). The collected samples were stored at 4 °C. Ten walnuts with the similar size of each sample were selected randomly for further experiments.

### Isolation of bacteria from in-shell walnut surface

The ten in-shell walnuts were placed in a sterile erlenmeyer flask containing 500 mL of 1% (w/v) peptone water supplemented with 1% (v/v) Tween-80. Then the erlenmeyer flask was vigorously shaken for approximately 20 min to thoroughly wash the surface of the in-shell walnuts^14, 52^. Finally the suspension was filtered over a sterile polycarbonate membrane (0.22 μm pore size) held in a sterilized filtration device. The membranes containing the bacterial filtrates were placed in sterilized centrifuge tubes and then stored at −80 °C until the DNA extraction step. The initial bacteria on the in-shell walnut surface without pre-cultured were also detected. Aerobic plate counts (APC) were determined by the pour plating method, and colonies were recorded as CFU/mL^53^.

### Extraction of total DNAs

Total DNAs were directly extracted from samples using the standardization CTAB method^54^. Briefly, the samples were added to the lysate containing lysozyme and 1000 μL CTAB buffer. The total DNA was extracted with chloroform: isoamyl alcohol (24:1) and precipitated with isopropanol at −20 °C. Then the precipitation was washed twice with 75% ethanol and dissolved with ddH_2_O. Finally, 1 μL RNase A was added to digest RNA at 37 °C for 15 min. DNA concentration and purity were examined by electrophoresis on 1% agarose gels. According to the concentration, DNA was diluted to 1 ng/μL using sterile water.

### PCR amplification

The amplification of the V4 region of 16 S rRNA genes used the specific primer with the barcode, and the universal primer with a forward primer 515 F (5′-GTGCCAGCMGCCGCGGTAA-3′) and a reverse primer 806 R (5′-GGACTACHVGGGTWTCTAAT-3′)^55^. The PCR reaction was performed using Phusion® High-Fidelity PCR Master Mix with GC buffer (New England Biolabs, Ipswich, MA, USA) in a 30 μL total volume (15 μL phusion master mix, 6 μM primer, 10 μL gDNA, 2 μL H_2_O).

The PCR reaction steps were conducted with a denaturation step at 98 °C for 1 min, followed by 30 cycles (98 °C, 10 sec; 50 °C, 30 sec; 72 °C, 30 sec), then with a final extension step at 72 °C for 5 min. The products were detected by electrophoresis using 2% agarose gel, and samples with bright main strip between 400–450 bp were chosen for further experiments. The PCR products were mixed in equidensity ratios, and then the mixture PCR products were purified with Gene JET Gel Extraction Kit (Thermo Scientific).

### High-throughput sequencing analysis

Sequencing libraries were generated using NEB Next® Ultra™ DNA Library Prep Kit for Illumina (NEB, USA) following manufacturer’s recommendations. After assessed on the Qubit@ 2.0 Fluorometer (Thermo Scientific) and Agilent Bioanalyzer 2100 system, the library was subjected to sequencing using Illumina HiSeq. 2500 platform at Nevogene (Beijing, China).

The paired-end reads were merged by using FLASH and then assigned to each sample according to the unique barcodes. The raw tags generated with FLASH were filtered and analyzed using QIIME software package^56, 57^. All effective reads from each sample were initially clustered into operational taxonomic units (OTUs) with ≥97% similarity. The RDP classifier^58^ was used to annotate taxonomic information for representative sequences of each OTU. The in-house Perl scripts were used to analyze alpha- (within samples) and beta- (among samples) diversity.

## Electronic supplementary material

Supplementary Information File
