# Supplementary material for: Bacterial community diversity on in-shell walnut surfaces from six representative provinces in China
Source: Sci Rep. 2017 Aug 30;7:10054. doi: 10.1038/s41598-017-10138-y (PMC5577159; doi:10.1038/s41598-017-10138-y)
Supplement: Supplementary file 1 — Supplementary Information File [file 41598_2017_10138_MOESM1_ESM.pdf]

Bacterial community diversity on in-shell walnut surfaces from six representative provinces in China

Lihui Zhang[1], Shaojin Wang[1, 2*]

[1]College of Mechanical and Electronic Engineering, Northwest A&F University, Yangling, Shaanxi 712100, China.[2] Department of Biological Systems Engineering, Washington State University, Pullman, WA 99164-6120, USA. Correspondence and requests for materials should be addressed to S. -J. W. (email: shaojinwang@nwsuaf.edu.cn)

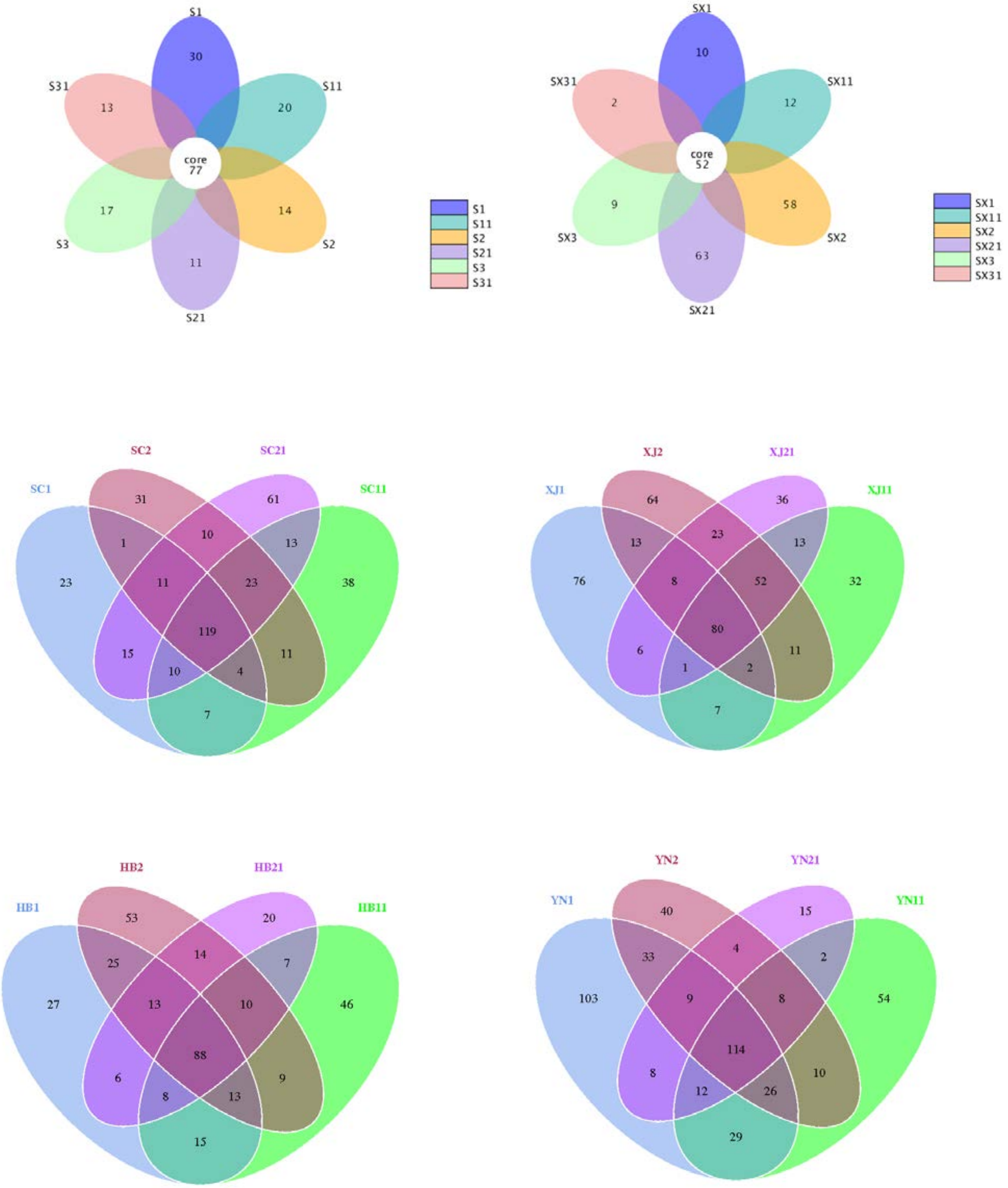

Fig. S1. Venn and flower plots of the samples within the same province.
